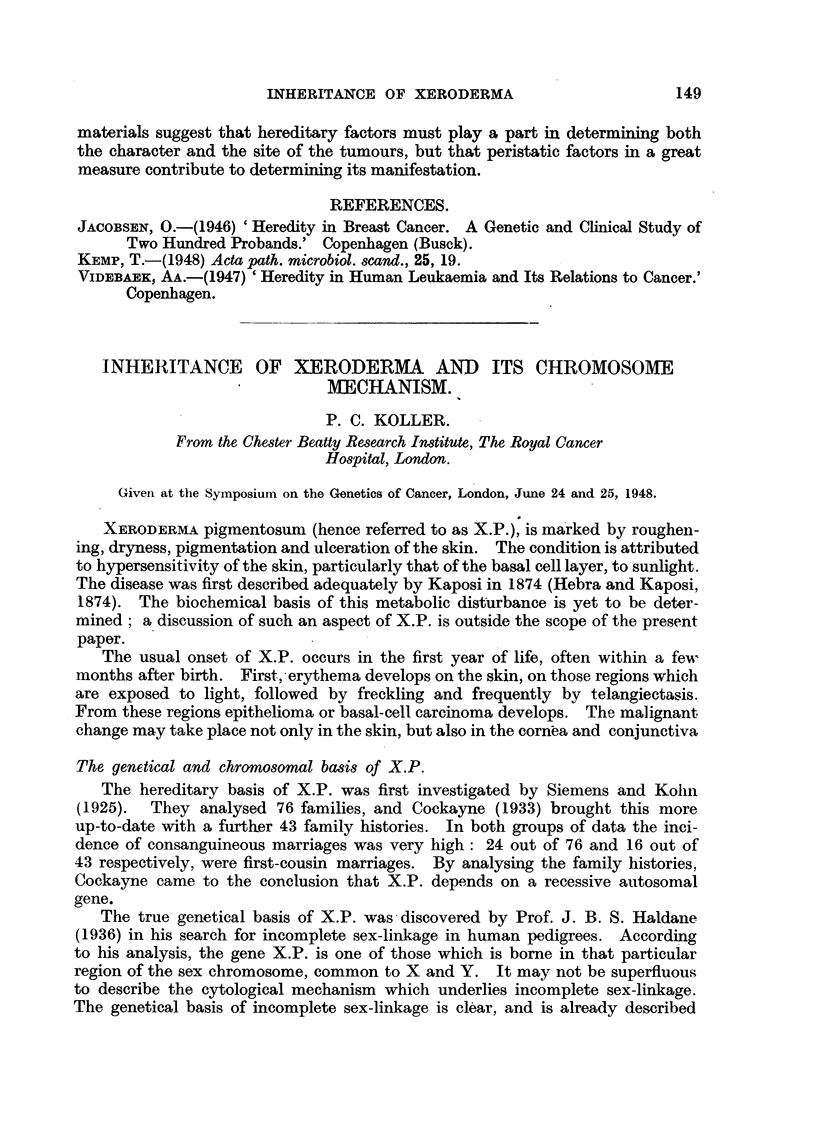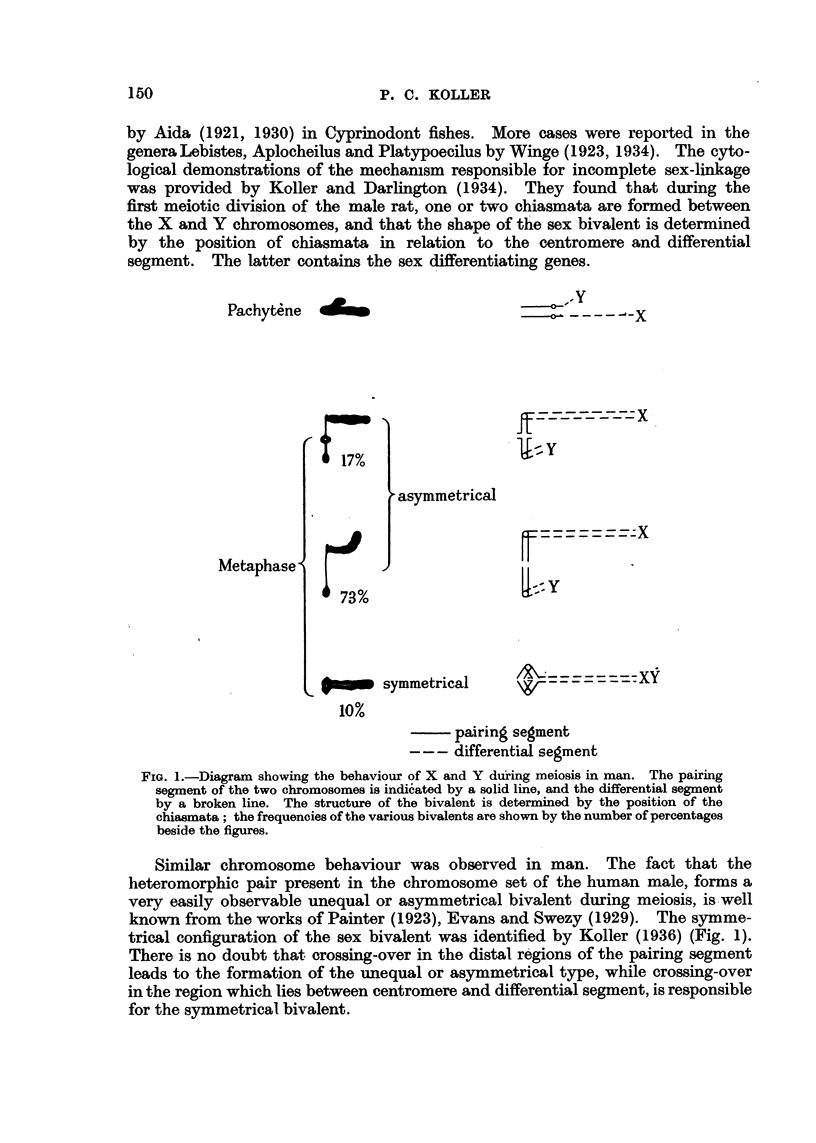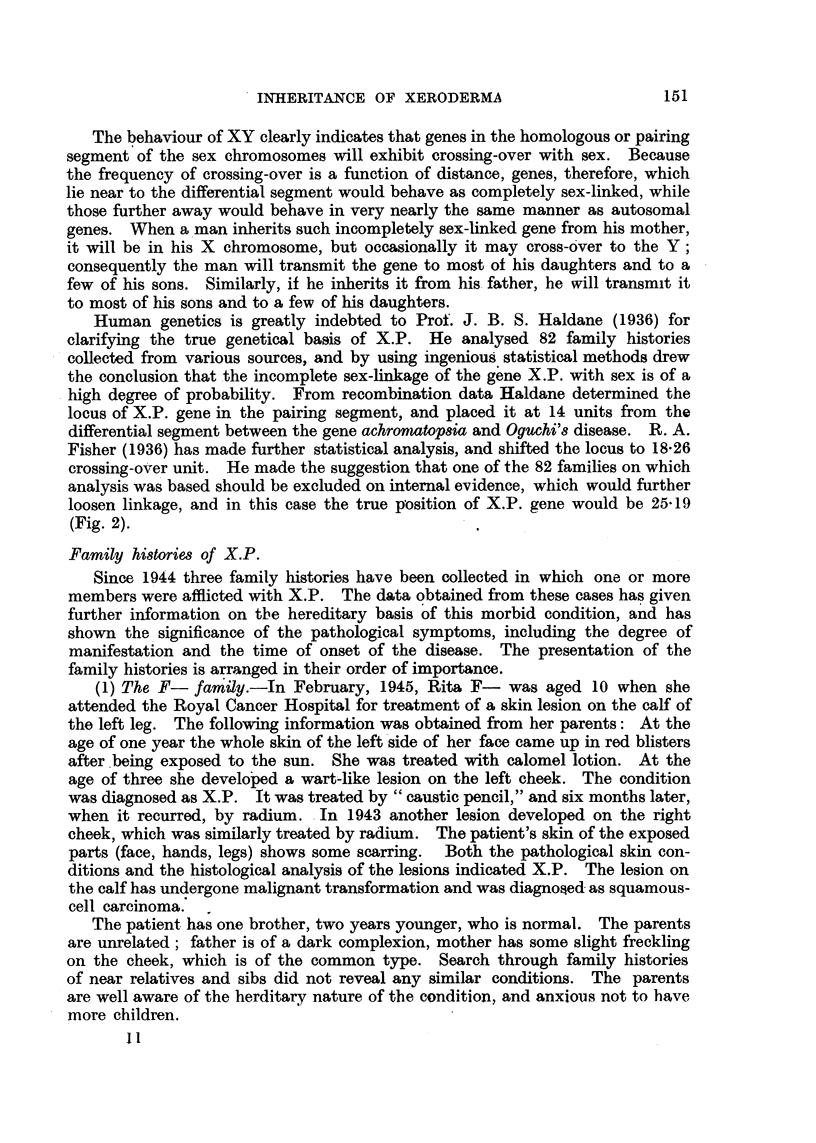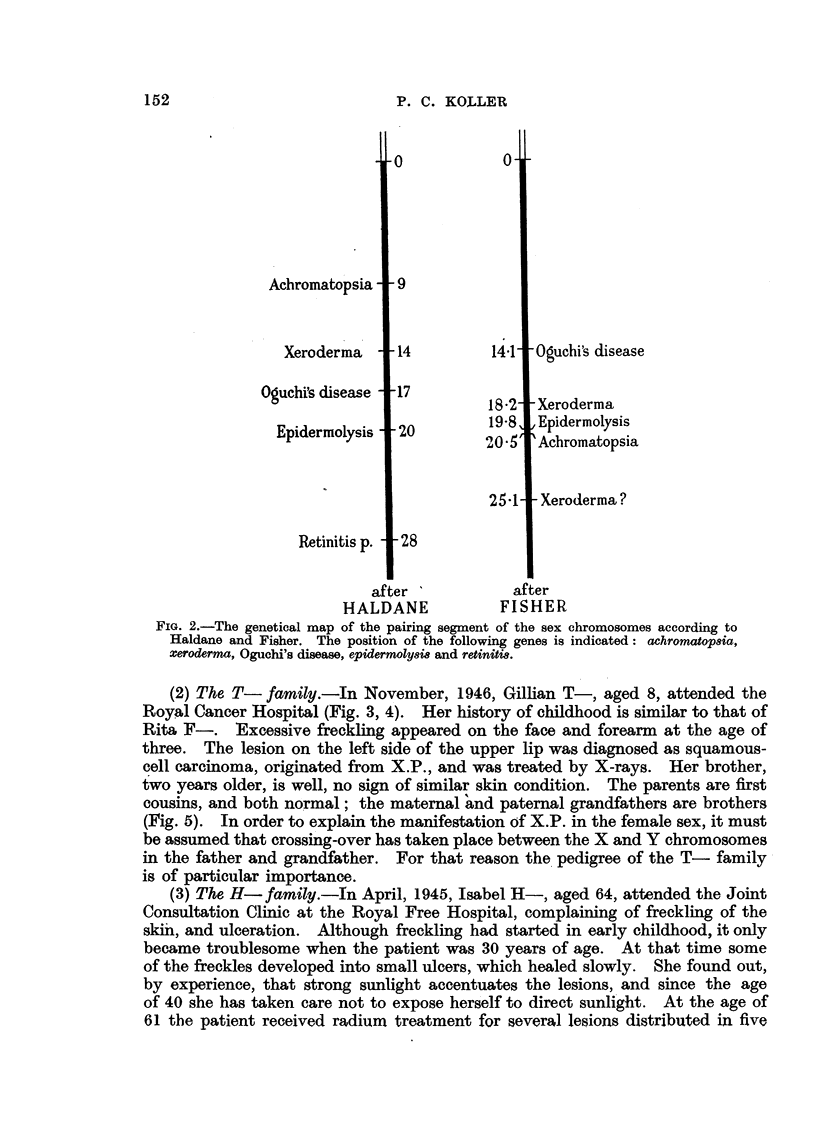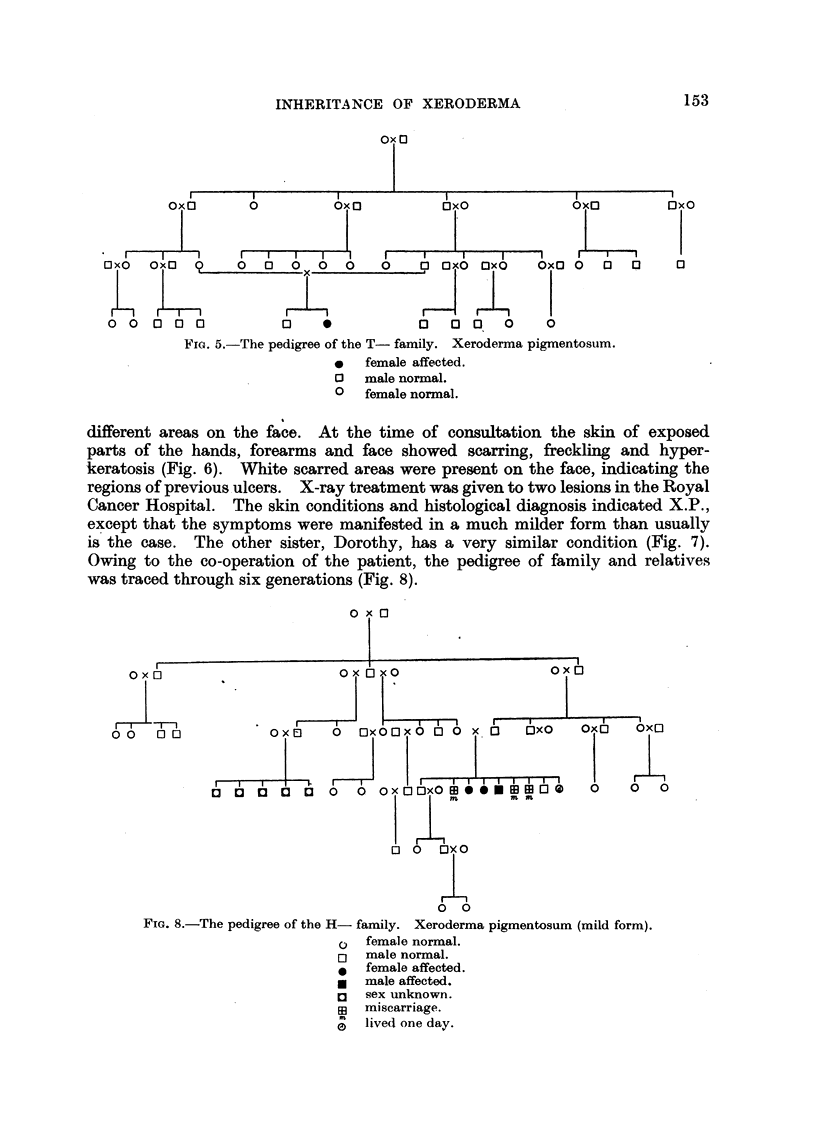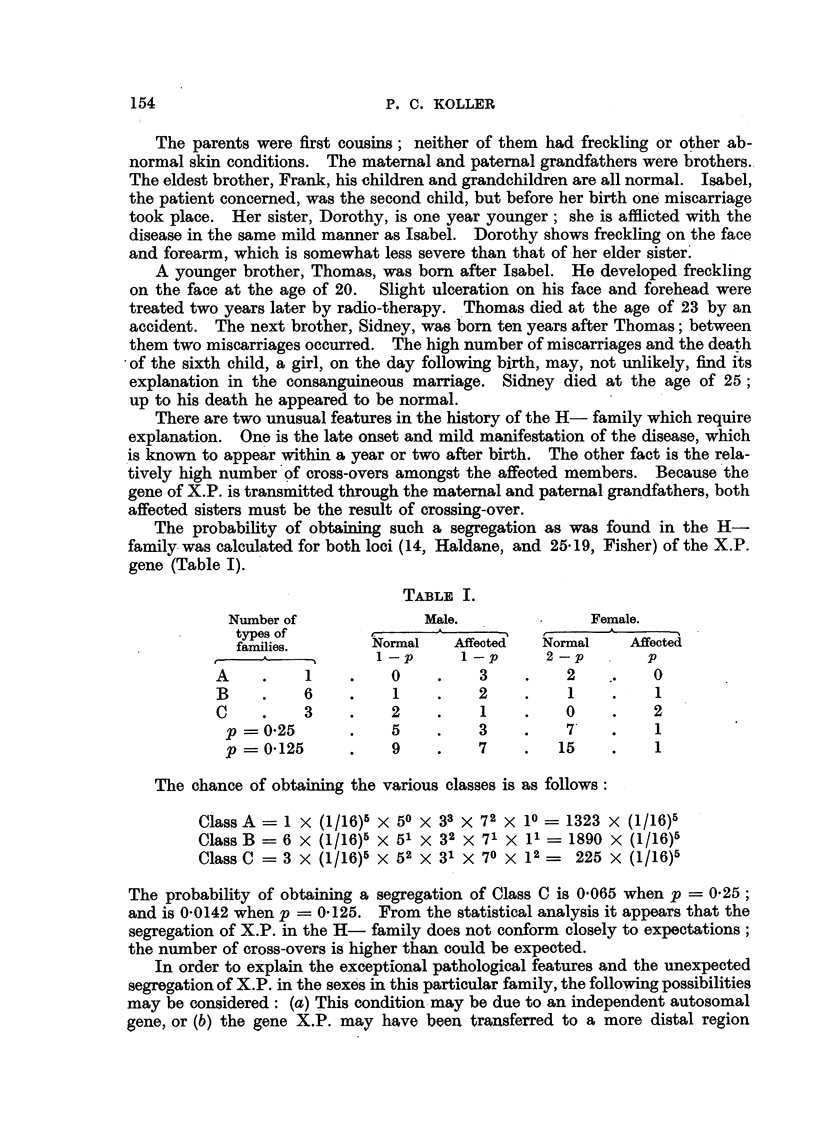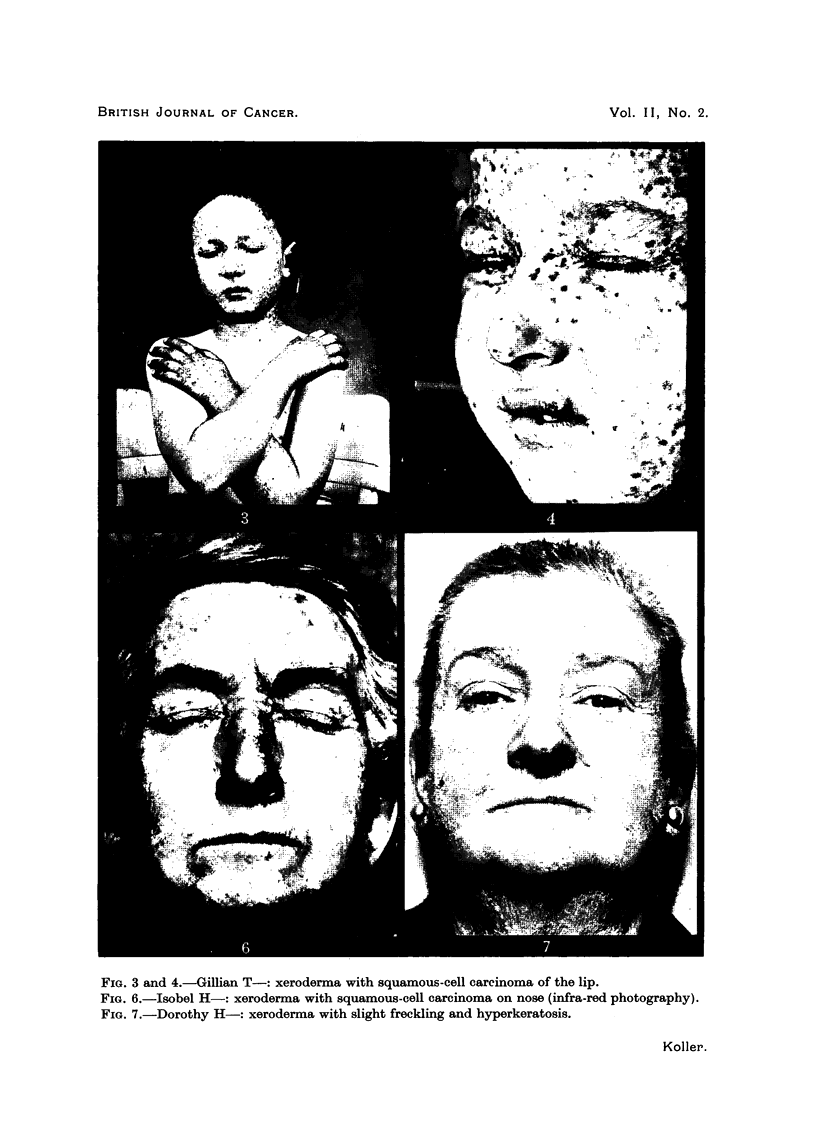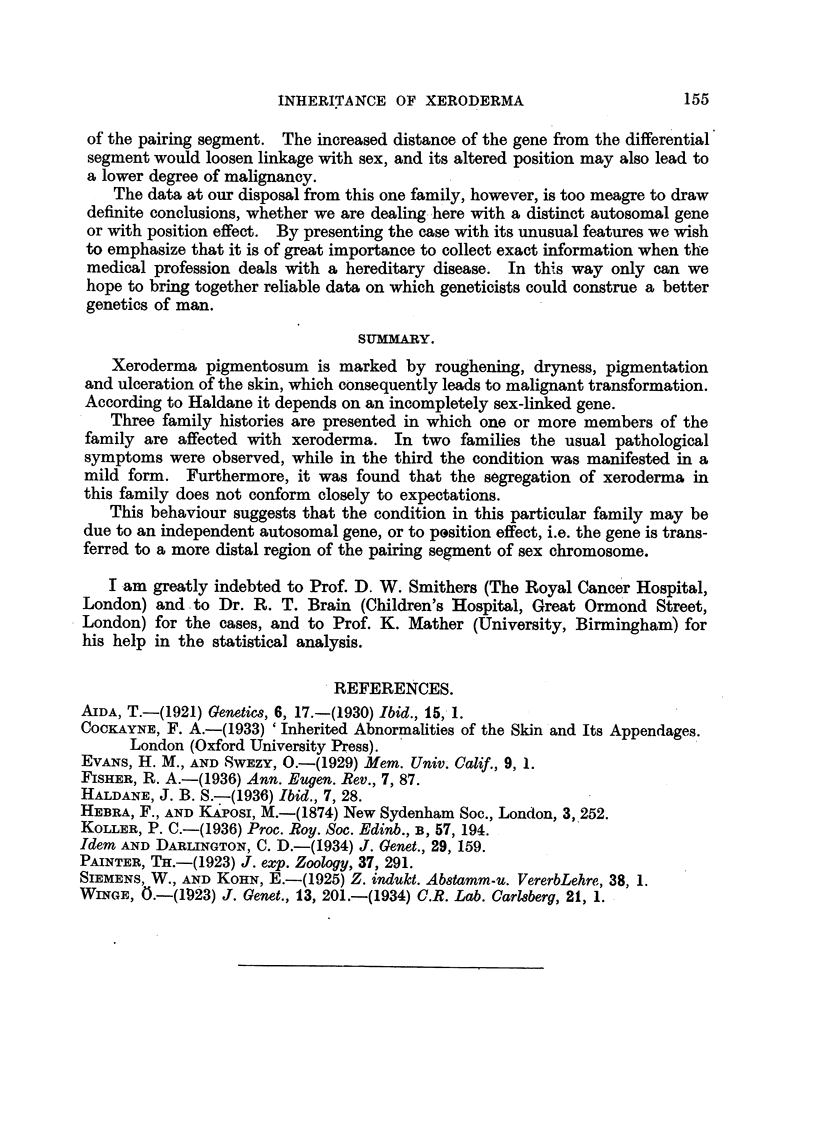# Inheritance of Xeroderma and its Chromosome Mechanism

**DOI:** 10.1038/bjc.1948.22

**Published:** 1948-06

**Authors:** P. C. Koller

## Abstract

**Images:**


### INHERITANCE OF XERODERMA AND ITS CHROMOSOME MECHANISM.

P. C. KOLLER.

*From the Chester Beatty Research Institute, The Royal Cancer Hospital, London.*

Given at the Symposium on the Genetics of Cancer, London, June 24 and 25, 1948.

XERODERMA pigmentosum (hence referred to as X.P.), is marked by roughening, dryness, pigmentation and ulceration of the skin. The condition is attributed to hypersensitivity of the skin, particularly that of the basal cell layer, to sunlight. The disease was first described adequately by Kaposi in 1874 (Hebra and Kaposi, 1874). The biochemical basis of this metabolic disturbance is yet to be determined ; a discussion of such an aspect of X.P. is outside the scope of the present paper.

The usual onset of X.P. occurs in the first year of life, often within a few months after birth. First, erythema develops on the skin, on those regions which are exposed to light, followed by freckling and frequently by telangiectasis. From these regions epithelioma or basal-cell carcinoma develops. The malignant change may take place not only in the skin, but also in the cornea and conjunctiva

*The genetical and chromosomal basis of X.P.*

The hereditary basis of X.P. was first investigated by Siemens and Kohn (1925). They analysed 76 families, and Cockayne (1933) brought this more up-to-date with a further 43 family histories. In both groups of data the incidence of consanguineous marriages was very high : 24 out of 76 and 16 out of 43 respectively, were first-cousin marriages. By analysing the family histories, Cockayne came to the conclusion that X.P. depends on a recessive autosomal gene.

The true genetical basis of X.P. was discovered by Prof. J. B. S. Haldane (1936) in his search for incomplete sex-linkage in human pedigrees. According to his analysis, the gene X.P. is one of those which is borne in that particular region of the sex chromosome, common to X and Y. It may not be superfluous to describe the cytological mechanism which underlies incomplete sex-linkage. The genetical basis of incomplete sex-linkage is clear, and is already described by Aida (1921, 1930) in Cyprinodont fishes. More cases were reported in the genera Lebistes, Aplocheilus and Platypoecilus by Winge (1923, 1934). The cytological demonstrations of the mechanism responsible for incomplete sex-linkage was provided by Koller and Darlington (1934). They found that during the first meiotic division of the male rat, one or two chiasmata are formed between the X and Y chromosomes, and that the shape of the sex bivalent is determined by the position of chiasmata in relation to the centromere and differential segment. The latter contains the sex differentiating genes.

Fig. 1.—Diagram showing the behaviour of X and Y during meiosis in man. The pairing segment of the two chromosomes is indicated by a solid line, and the differential segment by a broken line. The structure of the bivalent is determined by the position of the chiasmata ; the frequencies of the various bivalents are shown by the number of percentages beside the figures.

Similar chromosome behaviour was observed in man. The fact that the heteromorphic pair present in the chromosome set of the human male, forms a very easily observable unequal or asymmetrical bivalent during meiosis, is well known from the works of Painter (1923), Evans and Swezy (1929). The symmetrical configuration of the sex bivalent was identified by Koller (1936) (Fig. 1). There is no doubt that crossing-over in the distal regions of the pairing segment leads to the formation of the unequal or asymmetrical type, while crossing-over in the region which lies between centromere and differential segment, is responsible for the symmetrical bivalent.

The behaviour of XY clearly indicates that genes in the homologous or pairing segment of the sex chromosomes will exhibit crossing-over with sex. Because the frequency of crossing-over is a function of distance, genes, therefore, which lie near to the differential segment would behave as completely sex-linked, while those further away would behave in very nearly the same manner as autosomal genes. When a man inherits such incompletely sex-linked gene from his mother, it will be in his X chromosome, but occasionally it may cross-over to the Y; consequently the man will transmit the gene to most of his daughters and to a few of his sons. Similarly, if he inherits it from his father, he will transmit it to most of his sons and to a few of his daughters.

Human genetics is greatly indebted to Prof. J. B. S. Haldane (1936) for clarifying the true genetical basis of X.P. He analysed 82 family histories collected from various sources, and by using ingenious statistical methods drew the conclusion that the incomplete sex-linkage of the gene X.P. with sex is of a high degree of probability. From recombination data Haldane determined the locus of X.P. gene in the pairing segment, and placed it at 14 units from the differential segment between the gene *achromatopsia* and *Oguchi's* disease. R. A. Fisher (1936) has made further statistical analysis, and shifted the locus to 18·26 crossing-over unit. He made the suggestion that one of the 82 families on which analysis was based should be excluded on internal evidence, which would further loosen linkage, and in this case the true position of X.P. gene would be 25·19 (Fig. 2).

*Family histories of X.P.*

Since 1944 three family histories have been collected in which one or more members were afflicted with X.P. The data obtained from these cases has given further information on the hereditary basis of this morbid condition, and has shown the significance of the pathological symptoms, including the degree of manifestation and the time of onset of the disease. The presentation of the family histories is arranged in their order of importance.

(1) *The F— family.*—In February, 1945, Rita F— was aged 10 when she attended the Royal Cancer Hospital for treatment of a skin lesion on the calf of the left leg. The following information was obtained from her parents: At the age of one year the whole skin of the left side of her face came up in red blisters after being exposed to the sun. She was treated with calomel lotion. At the age of three she developed a wart-like lesion on the left cheek. The condition was diagnosed as X.P. It was treated by "caustic pencil," and six months later, when it recurred, by radium. In 1943 another lesion developed on the right cheek, which was similarly treated by radium. The patient's skin of the exposed parts (face, hands, legs) shows some scarring. Both the pathological skin conditions and the histological analysis of the lesions indicated X.P. The lesion on the calf has undergone malignant transformation and was diagnosed as squamous-cell carcinoma.

The patient has one brother, two years younger, who is normal. The parents are unrelated; father is of a dark complexion, mother has some slight freckling on the cheek, which is of the common type. Search through family histories of near relatives and sibs did not reveal any similar conditions. The parents are well aware of the herditary nature of the condition, and anxious not to have more children.

FIG. 2.—The genetical map of the pairing segment of the sex chromosomes according to Haldane and Fisher. The position of the following genes is indicated : *achromatopsia*, *xeroderma*, Oguchi's disease, *epidermolysis* and *retinitis*.

(2) *The T— family*.—In November, 1946, Gillian T—, aged 8, attended the Royal Cancer Hospital (Fig. 3, 4). Her history of childhood is similar to that of Rita F—. Excessive freckling appeared on the face and forearm at the age of three. The lesion on the left side of the upper lip was diagnosed as squamous-cell carcinoma, originated from X.P., and was treated by X-rays. Her brother, two years older, is well, no sign of similar skin condition. The parents are first cousins, and both normal ; the maternal and paternal grandfathers are brothers (Fig. 5). In order to explain the manifestation of X.P. in the female sex, it must be assumed that crossing-over has taken place between the X and Y chromosomes in the father and grandfather. For that reason the pedigree of the T— family is of particular importance.

(3) *The H— family*.—In April, 1945, Isabel H—, aged 64, attended the Joint Consultation Clinic at the Royal Free Hospital, complaining of freckling of the skin, and ulceration. Although freckling had started in early childhood, it only became troublesome when the patient was 30 years of age. At that time some of the freckles developed into small ulcers, which healed slowly. She found out, by experience, that strong sunlight accentuates the lesions, and since the age of 40 she has taken care not to expose herself to direct sunlight. At the age of 61 the patient received radium treatment for several lesions distributed in five

FIG. 5.—The pedigree of the T— family. Xeroderma pigmentosum.
● female affected.
□ male normal.
○ female normal.

different areas on the face. At the time of consultation the skin of exposed parts of the hands, forearms and face showed scarring, freckling and hyperkeratosis (Fig. 6). White scarred areas were present on the face, indicating the regions of previous ulcers. X-ray treatment was given to two lesions in the Royal Cancer Hospital. The skin conditions and histological diagnosis indicated X.P., except that the symptoms were manifested in a much milder form than usually is the case. The other sister, Dorothy, has a very similar condition (Fig. 7). Owing to the co-operation of the patient, the pedigree of family and relatives was traced through six generations (Fig. 8).

FIG. 8.—The pedigree of the H— family. Xeroderma pigmentosum (mild form).

○ female normal.

□ male normal.

● female affected.

■ male affected.

◘ sex unknown.

⊞ (m) miscarriage.

⊚ lived one day.

The parents were first cousins; neither of them had freckling or other abnormal skin conditions. The maternal and paternal grandfathers were brothers. The eldest brother, Frank, his children and grandchildren are all normal. Isabel, the patient concerned, was the second child, but before her birth one miscarriage took place. Her sister, Dorothy, is one year younger; she is afflicted with the disease in the same mild manner as Isabel. Dorothy shows freckling on the face and forearm, which is somewhat less severe than that of her elder sister.

A younger brother, Thomas, was born after Isabel. He developed freckling on the face at the age of 20. Slight ulceration on his face and forehead were treated two years later by radio-therapy. Thomas died at the age of 23 by an accident. The next brother, Sidney, was born ten years after Thomas; between them two miscarriages occurred. The high number of miscarriages and the death of the sixth child, a girl, on the day following birth, may, not unlikely, find its explanation in the consanguineous marriage. Sidney died at the age of 25; up to his death he appeared to be normal.

There are two unusual features in the history of the H— family which require explanation. One is the late onset and mild manifestation of the disease, which is known to appear within a year or two after birth. The other fact is the relatively high number of cross-overs amongst the affected members. Because the gene of X.P. is transmitted through the maternal and paternal grandfathers, both affected sisters must be the result of crossing-over.

The probability of obtaining such a segregation as was found in the H— family was calculated for both loci (14, Haldane, and 25·19, Fisher) of the X.P. gene (Table I).

TABLE I.

| Number of types of families. | | Male. | | Female. | |
|---|---|---|---|---|---|
| | | Normal $1-p$ | Affected $1-p$ | Normal $2-p$ | Affected $p$ |
| A | 1 | 0 | 3 | 2 | 0 |
| B | 6 | 1 | 2 | 1 | 1 |
| C | 3 | 2 | 1 | 0 | 2 |
| $p = 0{\cdot}25$ | | 5 | 3 | 7 | 1 |
| $p = 0{\cdot}125$ | | 9 | 7 | 15 | 1 |

The chance of obtaining the various classes is as follows:

$$\text{Class A} = 1 \times (1/16)^5 \times 5^0 \times 3^3 \times 7^2 \times 1^0 = 1323 \times (1/16)^5$$

$$\text{Class B} = 6 \times (1/16)^5 \times 5^1 \times 3^2 \times 7^1 \times 1^1 = 1890 \times (1/16)^5$$

$$\text{Class C} = 3 \times (1/16)^5 \times 5^2 \times 3^1 \times 7^0 \times 1^2 = 225 \times (1/16)^5$$

The probability of obtaining a segregation of Class C is 0·065 when $p = 0{\cdot}25$; and is 0·0142 when $p = 0{\cdot}125$. From the statistical analysis it appears that the segregation of X.P. in the H— family does not conform closely to expectations; the number of cross-overs is higher than could be expected.

In order to explain the exceptional pathological features and the unexpected segregation of X.P. in the sexes in this particular family, the following possibilities may be considered: (*a*) This condition may be due to an independent autosomal gene, or (*b*) the gene X.P. may have been transferred to a more distal region

3

4

6

7

Fig. 3 and 4.—Gillian T—: xeroderma with squamous-cell carcinoma of the lip.
Fig. 6.—Isobel H—: xeroderma with squamous-cell carcinoma on nose (infra-red photography).
Fig. 7.—Dorothy H—: xeroderma with slight freckling and hyperkeratosis.

of the pairing segment. The increased distance of the gene from the differential segment would loosen linkage with sex, and its altered position may also lead to a lower degree of malignancy.

The data at our disposal from this one family, however, is too meagre to draw definite conclusions, whether we are dealing here with a distinct autosomal gene or with position effect. By presenting the case with its unusual features we wish to emphasize that it is of great importance to collect exact information when the medical profession deals with a hereditary disease. In this way only can we hope to bring together reliable data on which geneticists could construe a better genetics of man.

SUMMARY.

Xeroderma pigmentosum is marked by roughening, dryness, pigmentation and ulceration of the skin, which consequently leads to malignant transformation. According to Haldane it depends on an incompletely sex-linked gene.

Three family histories are presented in which one or more members of the family are affected with xeroderma. In two families the usual pathological symptoms were observed, while in the third the condition was manifested in a mild form. Furthermore, it was found that the segregation of xeroderma in this family does not conform closely to expectations.

This behaviour suggests that the condition in this particular family may be due to an independent autosomal gene, or to position effect, i.e. the gene is transferred to a more distal region of the pairing segment of sex chromosome.

I am greatly indebted to Prof. D. W. Smithers (The Royal Cancer Hospital, London) and to Dr. R. T. Brain (Children's Hospital, Great Ormond Street, London) for the cases, and to Prof. K. Mather (University, Birmingham) for his help in the statistical analysis.

REFERENCES.

Aida, T.—(1921) *Genetics*, **6**, 17.—(1930) *Ibid.*, **15**, 1.
Cockayne, F. A.—(1933) 'Inherited Abnormalities of the Skin and Its Appendages. London (Oxford University Press).
Evans, H. M., and Swezy, O.—(1929) *Mem. Univ. Calif.*, **9**, 1.
Fisher, R. A.—(1936) *Ann. Eugen. Rev.*, **7**, 87.
Haldane, J. B. S.—(1936) *Ibid.*, **7**, 28.
Hebra, F., and Kaposi, M.—(1874) New Sydenham Soc., London, **3**, 252.
Koller, P. C.—(1936) *Proc. Roy. Soc. Edinb.*, b, **57**, 194.
*Idem* and Darlington, C. D.—(1934) *J. Genet.*, **29**, 159.
Painter, Th.—(1923) *J. exp. Zoology*, **37**, 291.
Siemens, W., and Kohn, E.—(1925) *Z. indukt. Abstamm-u. VererbLehre*, **38**, 1.
Winge, Ö.—(1923) *J. Genet.*, **13**, 201.—(1934) *C.R. Lab. Carlsberg*, **21**, 1.